# Distillate Flux Enhancement of Direct Contact Membrane Distillation Modules with Inserting Cross-Diagonal Carbon-Fiber Spacers

**DOI:** 10.3390/membranes11120973

**Published:** 2021-12-09

**Authors:** Chii-Dong Ho, Luke Chen, Jun-Wei Lim, Po-Hung Lin, Pin-Tsen Lu

**Affiliations:** 1 Department of Chemical and Materials Engineering, Tamkang University, Tamsui, New Taipei 251, Taiwan; ben225588@hotmail.com; 2 Department of Water Resources and Environmental Engineering, Tamkang University, Tamsui, New Taipei 251, Taiwan; luke@mail.tku.edu.tw (L.C.); polar87216@gmail.com (P.-T.L.); 3 Department of Fundamental and Applied Sciences, HICoE-Centre for Biofuel and Biochemical Research, Institute of Self-Sustainable Building, Universiti Teknologi PETRONAS, Seri Iskandar 32610, Perak Darul Ridzuan, Malaysia; junwei.lim@utp.edu.my

**Keywords:** hydrodynamic angles, temperature polarization effect, carbon-fiber spacers, pure water productivity

## Abstract

A new design of direct-contact membrane distillation (DCMD) modules with cross-diagonal carbon-fiber spacers of various hydrodynamic angles in flow channels to promote turbulence intensity was proposed to enhance pure water productivity. Attempts to reduce the temperature polarization coefficient were achieved by inserting cross-diagonal carbon-fiber spacers in channels, which create wakes and eddies in both heat and mass transfer behaviors to enhance the permeate flux enhancement. A simplified equation was formulated to obtain the theoretical predictions of heat transfer coefficients in the current DCMD device. The permeate fluxes and temperature distributions of both hot and cold feed streams are represented graphically with the inlet volumetric flow rate and inlet temperature of the hot saline feed stream as parameters. The higher distillate flux of countercurrent-flow operations for saline water desalination was accomplished as compared to the concurrent-flow operations of various hydrodynamic angles. The results show that the agreement between the theoretical predictions and experimental results is reasonably good. The effects of countercurrent-flow operations and inserting carbon fiber spacers have confirmed technical feasibility and device performance enhancement of up to 45%. The influences of operating and design parameters on the pure water productivity with the expense of energy consumption are also discussed.

## 1. Introduction

**Figure 1:**
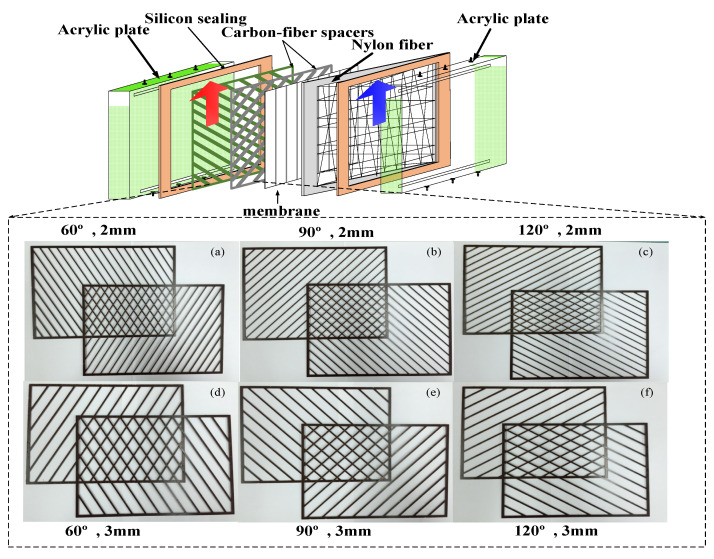
Fabrication structure and components of DCMD modules with cross-diagonal carbon-fiber spacers.

The advantages of membrane distillation (MD) systems to produce pure water by using low grade thermal energy [1] in remote villages or rural areas [2,3] are their simplicity and low operating cost. The DCMD module has been recognized as a potential technology for desalination, solution concentration and wastewater treatment [4]. The DCMD module is the separation process to vaporize the volatile species in the hot feed stream, and the permeate flux collected in the cold feed stream, in which the vapor pressure difference creates a driving force across the hydrophobic membrane surfaces [4] and yields high-purity water [5]. The temperature polarization effect [6] caused the thermal driving-force reduction and transmembrane permeate flux decrement in most previous studies of DCMD modules. Modification of hot fluid channels with various strategies diminish the thermal boundary layer and minimize the temperature polarization effect in DCMD modules, as shown in Figure 1. The mass transfer rate enhancement was accomplished by employing spacer-filled channels [7,8], filaments [9], and rough-surface channels [10], as well as inserting shell side baffles, wavy shape fibers and fibers with a gear-shaped cross section in hollow fiber modules [11] to increase permeate flux up to 30–300%. An alternative configuration improves the permeate flux by inserting cross-diagonal carbon-fiber spacers and adjusting hydrodynamic angles in channels of flat-plate DCMD modules in this study. Mathematical modeling equations were developed to analyze the device performance of the module by inserting cross-diagonal carbon-fiber spacers in channels, and thus, a correlated expression [12] of Nusselt numbers was obtained and validated by the experimental results. The optimal selection’s economic feasibility was investigated theoretically under both concurrent and countercurrent-flow operations. Our objective herein is to determine a permeate flux assessment under various operating conditions. The cross-diagonal carbon-fiber spacers act as eddy promoters to disturb the thermal boundary layer on the hot feed stream, and lead to a trade-off between permeate flux improvement and energy consumption increment. The suitable selection of hydrodynamic angles on the economic analysis for device performance enhancements was identified and explored.

## 2. Theoretical Modeling of DCMD Modules

Theoretical modeling equations of both heat and mass transfer behaviors for a DCMD module, as shown in Figure 1, were investigated to predict the permeate flux at the membrane/liquid interface of the hot saline feed stream, and diffused through porous hydrophobic membranes, and then condensed at the membrane/liquid interface in the cold stream as distillate flux. Three types of net-like cross-diagonal carbon-fiber spacers with various hydrodynamic angles were implemented in the hot feed side to promote the mass transfer rate, and comparisons of device performance were made between both modules with/without inserting cross-diagonal carbon-fiber spacers.

**Figure 2:**
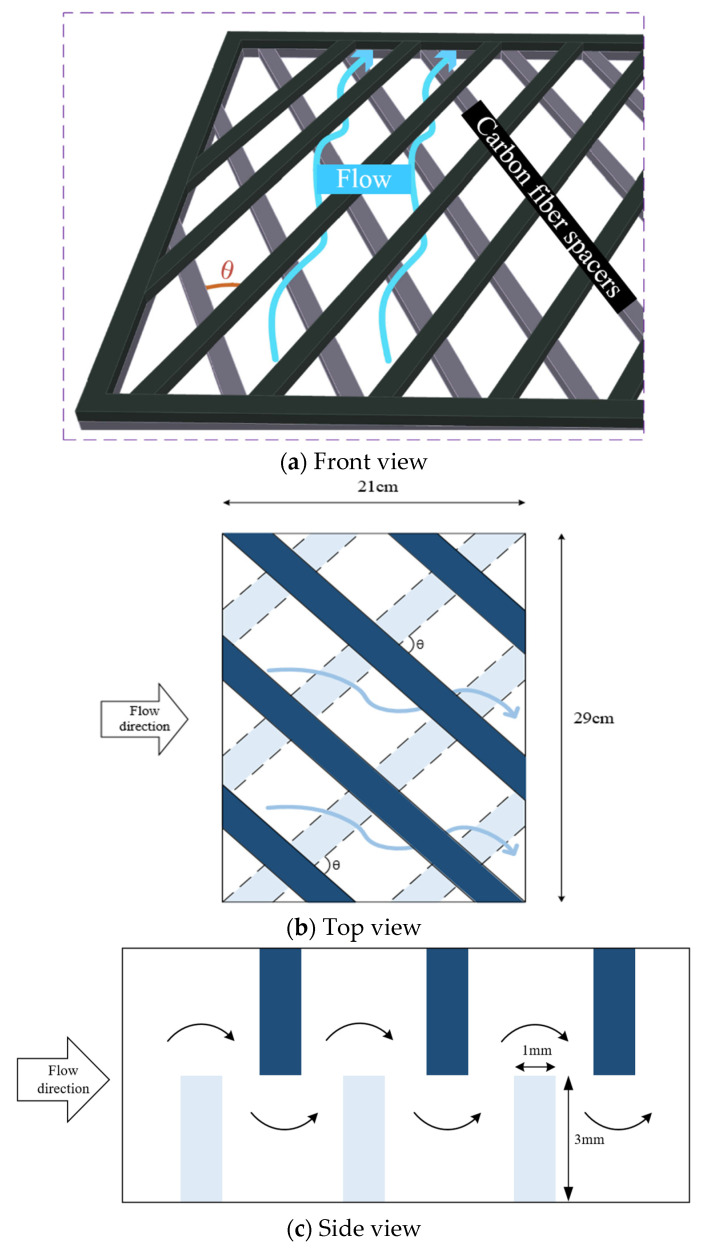
Saline flow streamlines in the spacer-filled flat-plate channel with hydrodynamic angle.

As hot saline water feed flows from grid to grid, a proportion of the feed stream will change direction by the hydrodynamic angle and follow a zigzag-like pathway, as seen in Figure 2.

Heat and mass transfer models were formulated according to the following assumptions:

(a) Steady-state operations;
(b) Physical properties of fluid, frame plates, and membrane are constants;
(c) Stagnant air within the membrane pore;
(d) Mass transfer by diffusion and heat transfer by conduction associated with latent heat through the hydrophobic membrane;
(e) No water transporting through the hydrophobic membrane;
(f) Good insulation on the entire circumference of modules.

**Figure 3:**
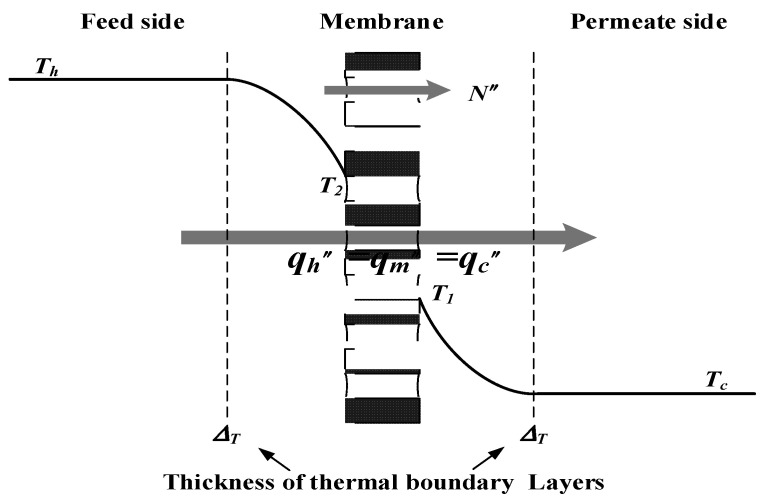
Schematic thermal boundary layers and temperature profiles of a DCMD module.

The non-isothermal process inside the DCMD module builds up the temperature gradient to enforce the permeate flux transferring across the hydrophobic membrane, which was condensed in the cold fluid stream as the pure water product. Theoretical modeling of both heat and mass transfer behaviors for a DCMD module was schematically illustrated in Figure 3. Mass-transfer modeling is needed to make balances of permeate flux by vapor diffusion, and then, the enthalpy flow conservation including heat conduction was formulated simultaneously in Equations (1)–(3) with the above assumptions as follows:

(1) $$q_{h}^{\prime \prime }=h_{h}(T_{h}-T_{2})—\mathrm{the}\;\mathrm{hot}\;\mathrm{saline}\;\mathrm{water}\;\mathrm{feed}\;\mathrm{region}\;$$

(2) $$q_{m}^{\prime \prime }=N^{\prime \prime }\lambda +k_{m}(T_{2}-T_{1})/\delta_{m}—\mathrm{the}\;\mathrm{membrane}\;\mathrm{region}$$

(3) $$q_{c}^{\prime \prime }=h_{c}(T_{1}-T_{c})—\mathrm{the}\;\mathrm{cooling}\;\mathrm{water}\;\mathrm{region}$$

where $N^{\prime \prime }\lambda$ is referred to as the latent heat of vaporization and $k_{m}(T_{2}-T_{1})/\delta_{m}$ is the conductive heat transfer, the thermal conductivity of the membrane $k_{m}$ can be determined by the thermal conductivities of vapor in the membrane pore $k_{g}$ and the solid membrane material $k_{s}$ is defined, following Warner [13], as:

(4) $$k_{m}=\varepsilon k_{g}+(1-\varepsilon )k_{s}$$

The membrane permeation coefficient ($c_{m}$) and the transmembrane saturation vapor pressure difference (ΔP) have been used extensively in mass transfer analysis of permeate flux for membrane distillation processes [2,14] as:

(5) $$N^{\prime \prime }=c_{m}\Delta P=c_{m}\left[P_{2}^{sat}(T_{2})-P_{1}^{sat}(T_{1})\right]=c_{m}{\frac{dP}{dT}|}_{T_{m}}(T_{2}-T_{1})=c_{m}\frac{P_{m}\lambda M_{w}}{RT_{m}^{2}}(T_{2}-T_{1})$$

where $P_{1}^{sat}$ and $P_{2}^{sat}$ are the saturated pressure of water vapor on both membrane surfaces, respectively.

The combinations of the heat flow and latent heat of Equation (2) yields the overall heat transfer coefficient of the membrane as follows:

(6) $$q_{m}^{\prime \prime }=N^{\prime \prime }\lambda +\frac{k_{m}}{\delta_{m}}(T_{2}-T_{1})=\left(c_{m}\frac{[a_{w}(1-x_{\mathrm{NaCl}})P_{2}+P_{1}]\lambda^{2}M_{w}}{2RT_{m}^{2}}+\frac{k_{m}}{\delta_{m}}\right)(T_{2}-T_{1})=H_{m}(T_{2}-T_{1})$$

where the membrane permeation coefficient $c_{w}$ is the addition of Knudsen diffusion and Poiseuille flow, $a_{w}=1-0.5x_{\mathrm{NaCl}}-10x_{\mathrm{NaCl}}^{2}$ is the activity coefficient [5] and the tortuosity τ=1/ε can be estimated using the porosity of the membrane [15].

The temperature polarization coefficient $T_{PC}$ is an indicator to indicate the extent of the thermal boundary-layer resistance which governs the distillate flux through the membrane. It is used to define as the ratio of membrane surface temperatures’ gradient to bulk temperatures’ gradient as follows:

(7) $$T_{PC}=(T_{2}-T_{1})/(T_{h}-T_{c})$$

Manipulating and solving Equations (1), (3), and (6) by equating all heat transfer regions under steady-state operations, say $q^{\prime \prime }=q_{h}^{\prime \prime }=q_{m}^{\prime \prime }=q_{c}^{\prime \prime }$ and neglecting the heat loss on the outside of the DCMD module leads to the following:

(8) $$T_{h}=T_{2}+\frac{H_{m}}{h_{h}}\left(T_{2}-T_{1}\right)$$

(9) $$T_{c}=T_{1}-\frac{H_{m}}{h_{c}}\left(T_{2}-T_{1}\right)$$

Equation (9) is subtracted from Equation (8) to give:

(10) $$T_{h}-T_{c}=\left(T_{2}-T_{1}\right)+\frac{H_{m}}{h_{h}}\left(T_{2}-T_{1}\right)+\frac{H_{m}}{h_{c}}\left(T_{2}-T_{1}\right)=\left(1+\frac{H_{m}}{h_{h}}+\frac{H_{m}}{h_{c}}\right)\left(T_{2}-T_{1}\right)$$

Moreover, thus an alternative form of Equation (7) for $T_{PC}$ expressed in terms of heat transfer coefficients leads to the following:

(11) $$T_{PC}=\frac{h_{h}h_{c}}{h_{h}h_{c}+h_{h}H_{m}+h_{c}H_{m}}$$

**Figure 4:**
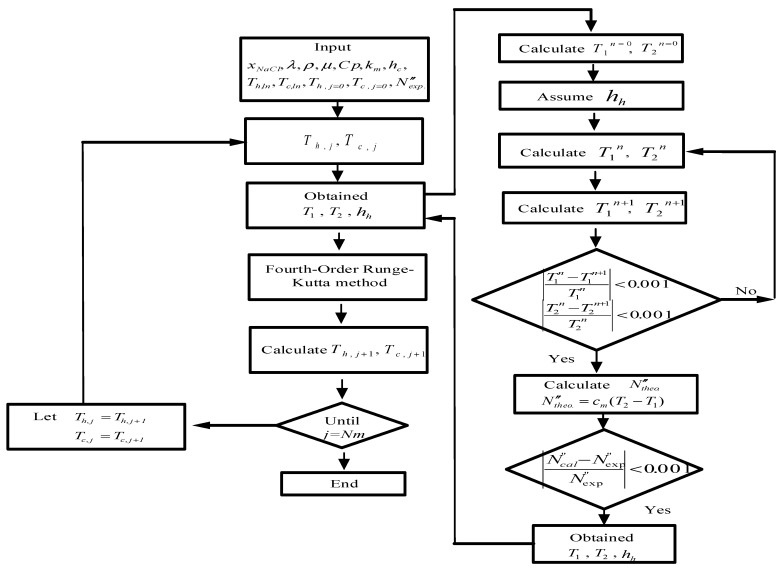
Flow chart for solving membrane surface temperatures and heat transfer coefficients.

The procedure for calculating of theoretical values of both membrane surface temperatures ($T_{1}$ and $T_{2}$) and the heat transfer coefficient will be described as follows. First, with the given operation conditions, the heat transfer coefficient is determined from Equations (8) and (9). Next, with the known inlet and outlet temperatures of both hot and cold streams, a temporary value of $T_{1}$ (or $T_{2}$) is estimated from Equation (8) once $T_{2}$ (or $T_{1}$) is assumed in Equation (9). Further, the convective heat transfer coefficient is calculated from Equation (5), with this calculated value of the convective heat transfer coefficient, new values of $T_{1}$ and $T_{2}$ are then recalculated from Equations (8) and (9). If the calculated values of $T_{1}$ and $T_{2}$ are different from the assumed values, continuous calculation by iteration is needed until the last assumed values of membrane surface temperatures meet the finally calculated values within a given convergence tolerance, as shown on the right-hand side of Figure 4.

**Figure 5:**
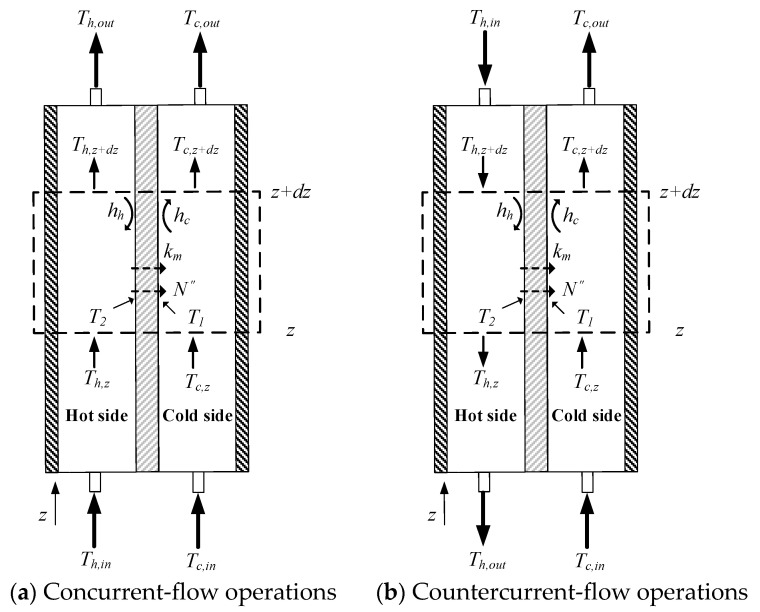
The energy balance made within a finite fluid element.

The one-dimensional modeling equations of the energy balances were obtained by making the energy-flow diagram presented in a finite fluid element, as shown in Figure 5, to solve the longitudinal temperature distributions of both hot and cold feed streams as:

(12) $$\frac{dT_{h}}{dz}=\frac{-q^{\prime \prime }W}{Q_{h}\rho_{h}C_{p,h}}=\frac{-W}{Q_{h}\rho_{h}C_{p,h}}H_{m}T_{PC}(T_{h}-T_{c})$$

(13) $$\frac{dT_{c}}{dz}=\frac{q^{\prime \prime }W}{Q_{c}\rho_{c}C_{p,c}}=\frac{W}{Q_{c}\rho_{c}C_{p,c}}H_{m}T_{PC}(T_{h}-T_{c})—\mathrm{concurrent}-\mathrm{flow}\;\mathrm{operations}$$

(14) $$\frac{dT_{c}}{dz}=\frac{-q^{\prime \prime }W}{Q_{c}\rho_{c}C_{p,c}}=\frac{-W}{Q_{c}\rho_{c}C_{p,c}}H_{m}T_{PC}(T_{h}-T_{c})—\mathrm{countercurrent}-\mathrm{flow}\;\mathrm{operations}$$

The temperature distributions of both hot and cold feed streams were solved in the above two simultaneous ordinary differential equations of Equations (12) and (13) for concurrent-flow operation (or Equation (14) for countercurrent-flow operation) with the use of the estimated convective heat transfer coefficients, and calculated iteratively in the left-hand side of Figure 4 by marching the fourth-order Runge–Kutta method numerically along the length of the DCMD module, and thus, the theoretical permeate flux and permeate flux enhancement were obtained. The temperature distributions were predicted theoretically not only in the hot/cold bulk flows and on the membrane surfaces of both hot and cold feed streams under concurrent- and countercurrent-flow operations, respectively. Comparisons were made between the channel with cross-diagonal carbon-fiber spacers and the device with the empty channel.

## 3. Experimental Apparatus and Procedures

**Figure 6:**
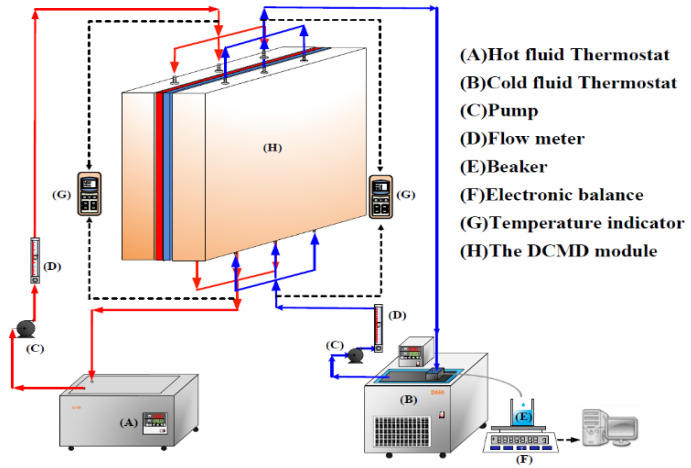
Schematic diagram of the experimental apparatus for the DCMD system.

A detailed configuration schematic of an acrylic parallel-plate channel of length 21 cm, width 29 cm, and 2 mm of each cold and hot stream is illustrated in Figure 6. The hydrophobic membrane surfaces were supported by inserting cross-diagonal carbon-fiber spacers and by winding a 0.1 mm nylon fiber in the hot saline and cold feed sides, respectively, to prevent the membrane bending and wrinkling.

**Figure 7:**
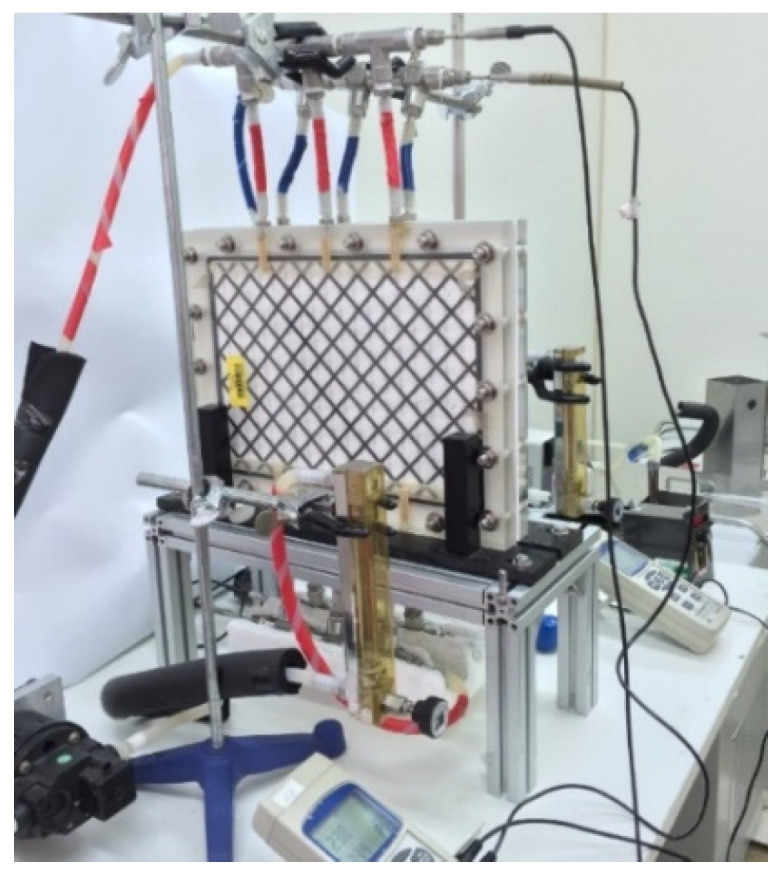
A photo of the experimental setup.

A commercial membrane, made of polytetrafluoroethylene (PTFE) supported by a polypropylene net (PP), was used in the experiments. The principal characteristics specified by the manufacturer (J020A330R, Toyo Roshi Kaisha, Ltd., Tokyo, Japan) are nominal pore size of 0.2 µm, porosity of 0.72, and thickness of 130 µm. PTFE is a hydrophobic membrane of many unique properties with four components, i.e., calcium fluoride, hydrofluoric acid, chloroform and water. The average molecular weight of the membranes ranges from 400,000 to 9,000,000. The manufacturing process involves the synthesis and polymerization of tetrafluoroethylene (TFE) with a series of chemical reactions to create the final product PTFE membrane which is exceptionally resistant to corrosion. The pore size and porosity of the hydrophobic composite membrane made of PTFE/PP will affect the permeation flux. However, the conductance of the distillate flux collected should be monitored and measured during the experimental runs, and it was less than 1.5 μs/cm in the present work. The cross-diagonal carbon-fiber spacers were implemented with various hydrodynamic angles in channels to generate vortices around those net-like carbon-fiber open slots, as shown in Figure 1 and Figure 2. The structure of the spacer is shown by the picture in Figure 7. A silicon rubber with thickness of 2 mm was glued on the acrylic plate to build up a spacer channel and also to prevent leakage.

Experimental runs were conducted under various operating conditions to study the device performances of permeate fluxes for two modules with/without inserting cross-diagonal carbon-fiber spacers in channels. The artificial saline water of 3.5 wt% NaCl was prepared by adding inorganic salts NaCl into distilled water. The experiments were operated by controlling various flow rates (0.1, 0.2, 0.3, 0.4 L/min) for various inlet hot saline temperatures (40, 45, 50, 55 °C) and 25 °C of the cold stream, and read by thermocouples (TM-946, Lutron, New Taipei, Taiwan). The temperatures of both streams were regulated by the thermostat (Water Bath G-50, DENG YNG, Taiwan) and the thermostat (Water Bath D650, DENG YNG, Taiwan), respectively. The distillate flux condensed in the cold side was then collected and weighed using an electronic balance (XS 4250C, Precisa Gravimetrics AG, Dietikon, Switzerland) to measure the distillate flux and recorded on the PC.

**Figure 8:**
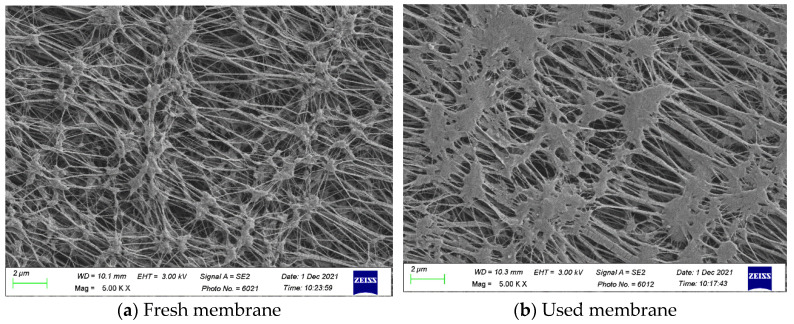
SEM micrographs of the fresh membrane and the used membrane after experimental runs. (**a**) The fresh membrane; (**b**) the used membrane.

Figure 8 presents the SEM micrographs of the fresh membrane and the used membrane after experimental runs. The SEM images indicated that some salts were stuck on the membrane surface, but most of porous channels were not jammed by the salts.

## 4. Flux Enhancement Factor and Power Consumption Increment

The enhancement factor $\alpha^{E}$ depends on various hydrodynamic angles, compared to the empty channel, and was correlated to calculate the augmented convective heat transfer coefficients in DCMD modules with implementing cross-diagonal carbon-fiber spacers [16] as:

(15) $$\alpha^{E}=Nu^{E}/Nu_{lam}$$

where

(16) $$Nu^{E}=\frac{h_{h}D_{h,h}}{k}—\mathrm{for}\;a\;\mathrm{module}\;\mathrm{with}\;\mathrm{inserted}\;\mathrm{cross}-\mathrm{diagonal}\;\mathrm{carbon}-\mathrm{fiber}\;\mathrm{spacers}$$

(17) Nulam=4.36+0.036RePr(Dh,h/L)1+0.011[RePr(Dh,h/L)]0.8—for a module with an empty channel

A better interpretation of both heat and mass transfer behaviors in the module with cross-diagonal carbon-fiber spacers could be described by a new method based on dimensional analysis of the Buckingham’s π theorem, which expresses the influence of eddies and vortices created by the turbulent flow due to the eddy promoter, and generates more turbulence intensity. The Nusselt number of flow channels with cross-diagonal carbon-fiber spacers can be related to dimensionless groups:

(18) $$Nu^{E}=f\;\left(\frac{W_{e}}{D_{h,h}},\sin\theta \right)$$

where $W_{e}$ and $D_{h,h}$ are the carbon-fiber spacer width and hydraulic diameter of the hot stream side, respectively. The average velocity [17] and equivalent hydraulic diameter of cold and hot stream sides are defined as follows:

(19) $${\bar{\nu }}_{h}=\frac{Q_{h}}{dD\varepsilon_{e}},{\bar{\nu }}_{c}=\frac{Q_{c}}{dW}$$

(20) $$D_{h,h}=\frac{4\varepsilon_{e}}{(2/d)+\left(1-\varepsilon_{e}\right)S_{vsp}},\;D_{h,c}=\frac{4dW}{2\left(d+W\right)}$$

(21) $$S_{vsp}=\frac{surface}{volume}=\frac{S_{sp}}{V_{sp}}=\frac{2(W_{e}+d_{p})}{W_{e}d_{p}}$$

(22) Reh=ρhv¯hdh,hμh, Rec=ρcv¯cdh,cμc

The power consumption increment is required due to inserting cross-diagonal carbon-fiber spacers into the saline water feed channel as eddy promoters. The friction losses to walls of both hot and cold streams were assumed to be significant, which were calculated in determining the power consumption by using Fanning friction factor $f_{F}$ [18]:

(23) $$H_{i}={\dot{m}}_{h}\ell w_{f,h}+{\dot{m}}_{c}\ell w_{f,c}=Q_{h}\rho_{h}\ell w_{f,h}+Q_{c}\rho_{c}\ell w_{f,c}\;i=\mathrm{carbon}\;\mathrm{fiber},\;\mathrm{empty}\;$$

(24) $$\ell w_{f,j}=\frac{2f_{F,j}{\bar{v}}_{j}^{2}L}{d_{h,i}},\;j=h,c$$

in which (β=d/W) [19]:

(25) fF,j=24(1−1.3553β+1.9467β 2−1.7012β 3+0.9564β 4−0.2537β 5)/Rej,j = h , c

The power consumption increment $I_{P}$ due to the friction losses in the conduits can be readily derived as follows:

(26) $$I_{C}=\frac{H_{carbon\;fiber}-H_{empty}}{H_{empty}}\times 100\%$$

## 5. Results and Discussion

**Figure 9:**
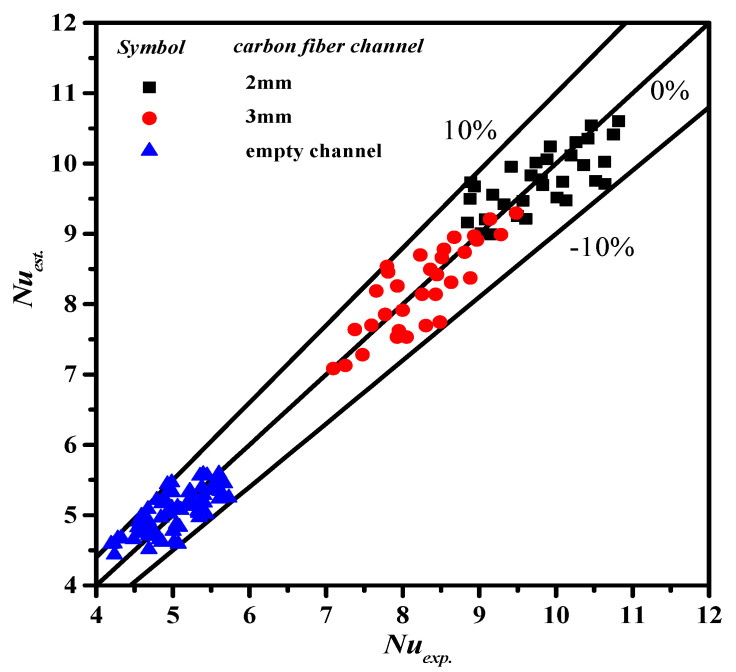
Comparison of estimated and experimental Nusselt numbers.

The estimated values of membrane surface temperatures and the convective heat transfer coefficients were obtained using a numerical flowchart in Figure 4, which were plugged into Equations (16) and (17) (Equation (18) for countercurrent-flow operations) in solving numerically by the fourth-order Runge–Kutta method of both bulk temperature distributions in hot/cold feed streams along the flowing direction of DCMD module. The theoretical predictions of permeate flux and permeate flux enhancement for the module with inserting cross-diagonal carbon-fiber spacers were thus obtained, and compared to the device with the empty channel. The experimental results with empty channel and 2 and 3 mm carbon-fiber slots were used to regress the correlation for the enhancement factor $\alpha^{E}$, as expressed in Equation (18). The resultant expression was determined via a regression analysis from curve-fitting with the squared correlation coefficient ($R^{2}=0.952$), as shown in Figure 9.

(27) $$\alpha^{E}=\frac{Nu^{E}}{Nu_{lam}}=3.163\exp{\left(\frac{W_{e}}{d_{h,h}}\right)}^{-0.766}\sin\theta^{-0.112}$$

**Figure 10:**
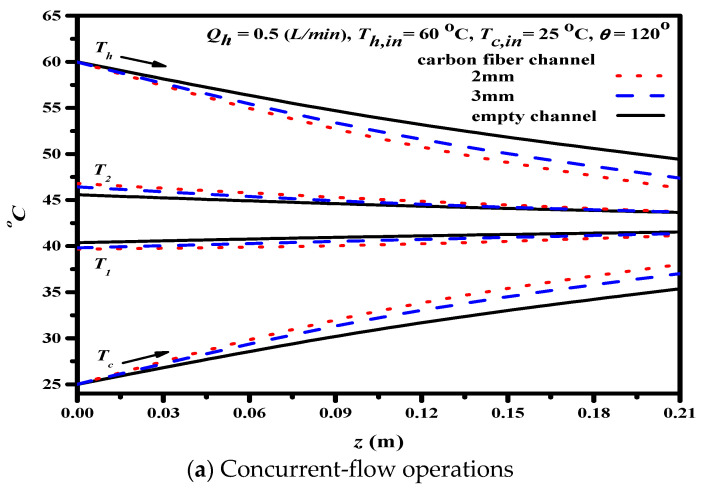

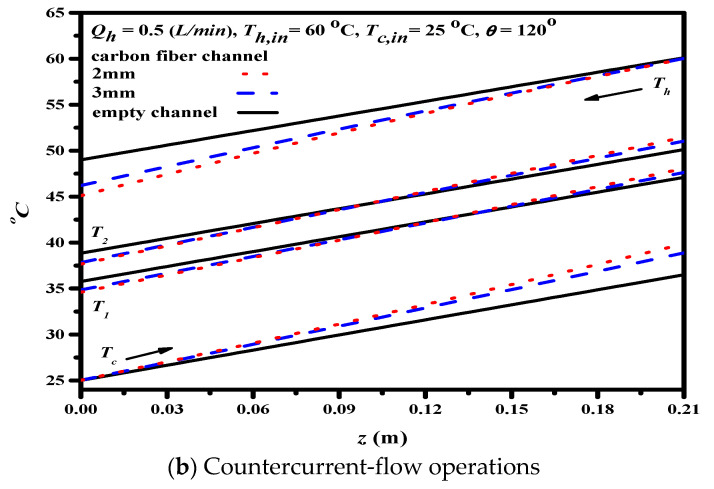
Effect of net-like cross-diagonal carbon-fiber spacers on temperature profiles.

The effect of the carbon-fiber spacers on the longitudinal temperature profiles of both hot and cold feed streams in the DCMD module was shown in Figure 10 with hydrodynamic angles of 120° as an illustration. The temperature profiles show that both membrane surface temperatures of both hot and cold feed streams with/without net-like cross-diagonal carbon-fiber spacers are considerably different for both concurrent- and countercurrent-flow operations.

The permeate flux is proportional to the temperature gradient between both membrane surface temperatures $T_{1}$ and $T_{2}$ in the DCMD system. The temperature gradient between both membrane surfaces is higher in the carbon-fiber spacers channel than that in the empty channel. Reduction of the temperature polarization effect was achieved using carbon-fiber spacers in the channel for a promising result investigated by computational simulation [20]. The more significant temperature gradient results in a larger heat flux of the device with net-like cross-diagonal carbon-fiber spacers; hence more permeate flux or pure water productivity was predicted, as indicated in Equation (5). One can find that the temperature gradient of the carbon-fiber spacer width of 2 mm is higher than that of the 3 mm spacer. The temperature gradient appears a nonuniform profile and tapers from the higher value at the entrance to the outlet in concurrent-flow operations during the comparatively uniform temperature gradient of the countercurrent-flow operations. The descending heat transfer rate and permeate flux along the channel for concurrent-flow operations is thus confirmed as compared to a higher heat transfer rate and permeate flux in countercurrent-flow operations.

**Figure 11:**
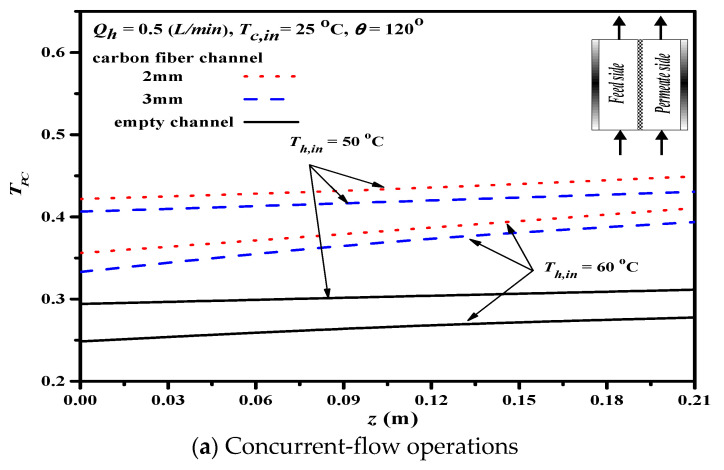

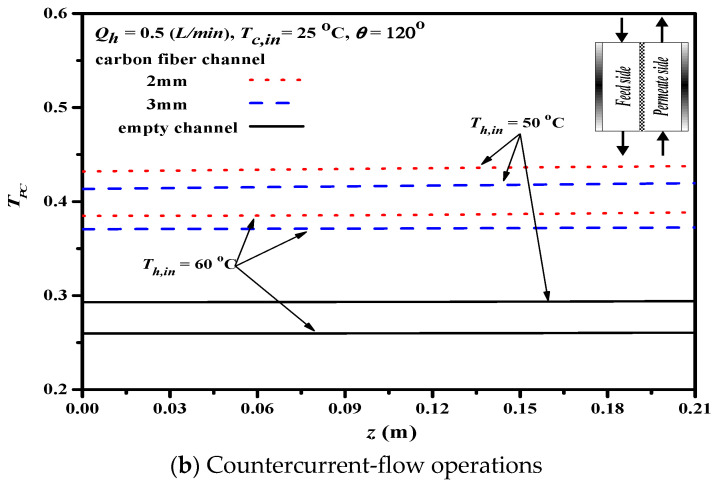
Effect of net-like cross-diagonal carbon-fiber spacers on $T_{PC}$.

The devices with net-like cross-diagonal carbon-fiber spacers were used for promoting eddies and for temperature polarization reduction. This study investigated and compared the effects of carbon-fiber spacer widths on temperature polarization, as depicted in Figure 11.

The theoretical predictions of $T_{PC}$ show that a higher inlet temperature of the hot saline feed stream leads to a lower $T_{PC}$ for both concurrent- and countercurrent-flow operations, because the higher permeate flux needs more latent heat of vaporization, which results in a lower temperature gradient across the membrane surface. Implementing cross-diagonal carbon-fiber spacers into the hot feed stream intensifies turbulence, reduces the thickness of thermal boundary layer at the membrane surface, and increases $T_{PC}$ (i.e., lower temperature polarization). The above procedure leads to the reduction in the thickness of thermal boundary-layer thickness and increment in heat transfer rate, compared to the module without the spacer. In addition, inserting 2 mm cross-diagonal carbon-fiber spacers does have a larger $T_{PC}$ value (a higher heat transfer rate) than the 3 mm cross-diagonal carbon-fiber spacers and the device with the empty channel, which could result from more net-like opening slots to induce turbulence. Furthermore, a higher $T_{PC}$ value (say less thermal resistance) was achieved in countercurrent-flow operations than that in the DCMD system’s concurrent-flow operations. Restated, the reduction of temperature polarization effect in decreasing the temperature gradient between the bulk stream and membrane surface, say the thinner thermal boundary layer with a larger $T_{PC}$ value, and thus, yields a higher permeate flux through the hydrophobic membrane.

The agreement between the experimental results and theoretical predictions is in good consistency, where the accuracy deviation of the theoretical predictions from the experimental results are within an acceptable range, as indicated in Table 1a,b with the definition as follows:

(28) $$E(\%)=\frac{1}{N}\sum_{i=1}^{N_{\exp}}\frac{\left|N_{theo}^{\prime \prime }-N_{\exp}^{\prime \prime }\right|}{N_{\exp}^{\prime \prime }}\times 100$$

**Figure 12:**
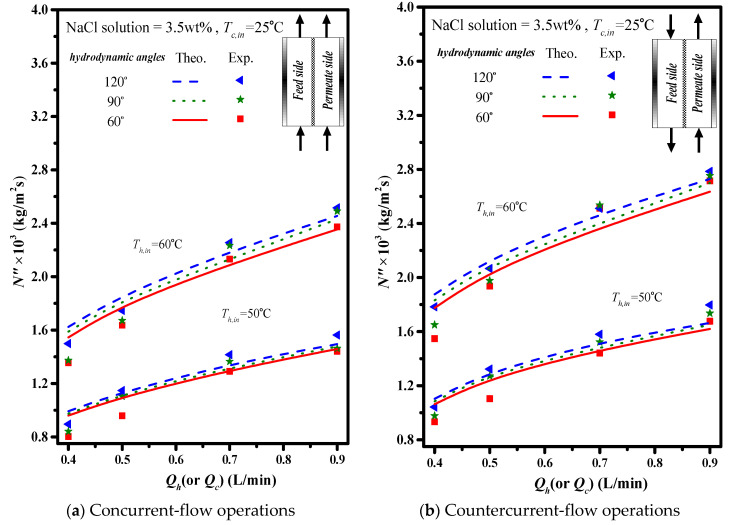
Effects of hydrodynamic angles and operation types on permeate flux (2 mm spacers).

Comparisons were made on theoretical predictions and experimental results of permeate fluxes between the empty channel and the channels with 2 mm cross-diagonal carbon-fiber spacers, as shown in Figure 12a,b as well as in Table 1a,b for illustrations. The results show that the permeate flux increases with the increase of the inlet volumetric flow rate, inlet saline temperature and hydrodynamic angles, and the extent of permeate flux increment is more significant in countercurrent-flow operations.

**Figure 13:**
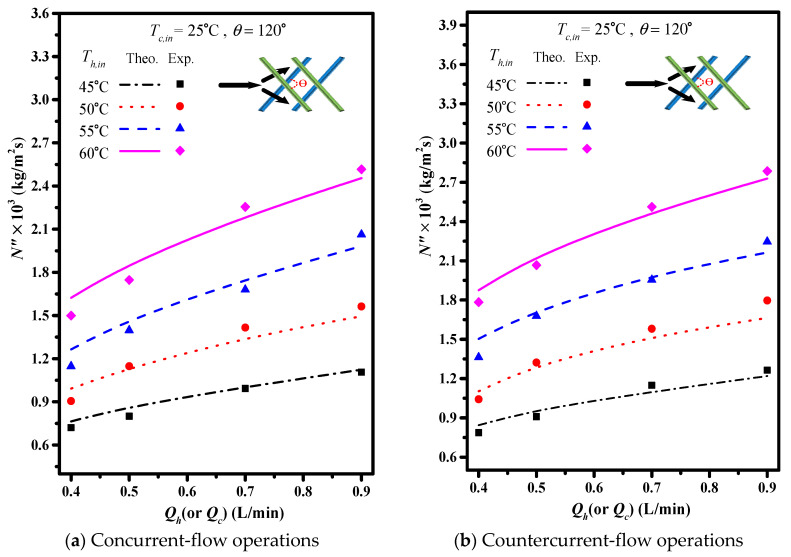
Effects of inlet saline temperatures on permeate flux (2 mm).

**Figure 14:**
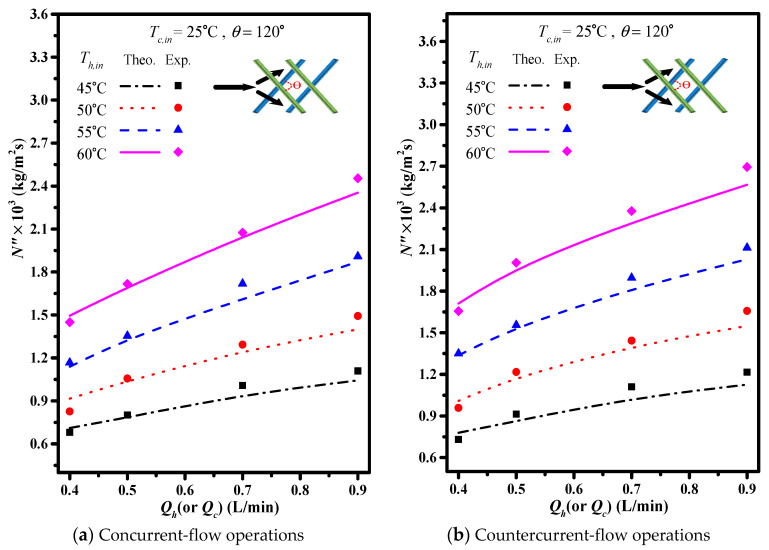
Effects of inlet saline temperatures on permeate flux (3 mm).

The theoretical predictions and experimental results of permeate flux were presented graphically in Figure 13 and Figure 14 for the empty channel and the channels with 2 mm and 3 mm inserting cross-diagonal carbon-fiber spacers, respectively. The permeate flux increases with the volumetric flow rate, hydrodynamic angle and the inlet saline temperature. Notice that the effect of hydrodynamic angle on the permeate flux concludes that more permeate flux with the use of the turbulence promoter by inserting cross-diagonal carbon-fiber spacers.

The improvement of the device performance $I_{E}$ from inserting the cross-diagonal carbon-fiber spacers is best illustrated by the percentage increase in the permeate flux compared to the device with an empty channel.

(29) $$I_{E}=\frac{N_{promoter}^{\prime \prime }-N_{empty}^{\prime \prime }}{N_{empty}^{\prime \prime }}\times 100\%$$

The permeate flux enhancements $I_{E}$ of the device with cross-diagonal carbon-fiber spacers in the hot saline stream were significant achievements under both concurrent- and countercurrent-flow operations, as demonstrated in Table 2a,b for 2 mm and 3 mm cross-diagonal carbon-fiber spacers, respectively. Both experimental results and the theoretical predictions were conducted in comparisons with various hydrodynamic angles, carbon-fiber spacer widths, inlet saline temperatures and inlet volumetric flow rate as parameters. Effects of hydrodynamic angle and the width of carbon-fiber spacer show that the permeate flux increases with the hydrodynamic angle but decreases with the spacer width. Overall, the performance of permeate flux is enhanced by inserting the cross-diagonal carbon-fiber spacers into the channel, which serves as eddy promoters in both concurrent- and countercurrent-flow operations. The results indicate that the permeate flux enhancement is up to 45%. Hence, the device with insertion of cross-diagonal carbon-fiber spacers may also be applied to the pressure-driven membrane distillation processes and water-treatment technologies.

**Figure 15:**
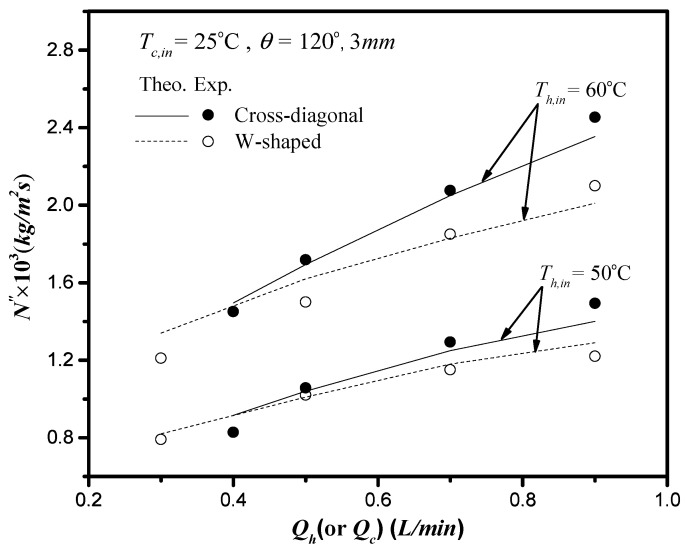
Comparisons of theoretical predictions and experimental results of permeate flux two type of carbon-fiber spacers (3 mm; concurrent-flow operations).

The present work extends the previous study except for inserting cross-diagonal carbon-fiber spacers instead of W-shaped carbon-fiber spacers [21]. The graphical representation for comparisons with theoretical predictions of the permeate flux obtained in the present study and W-shaped carbon-fiber spacers [21] illustrates why the present configurations of inserting cross-diagonal carbon-fiber spacers is preferred, presented in Figure 15 for concurrent-flow operations.

**Figure 16:**
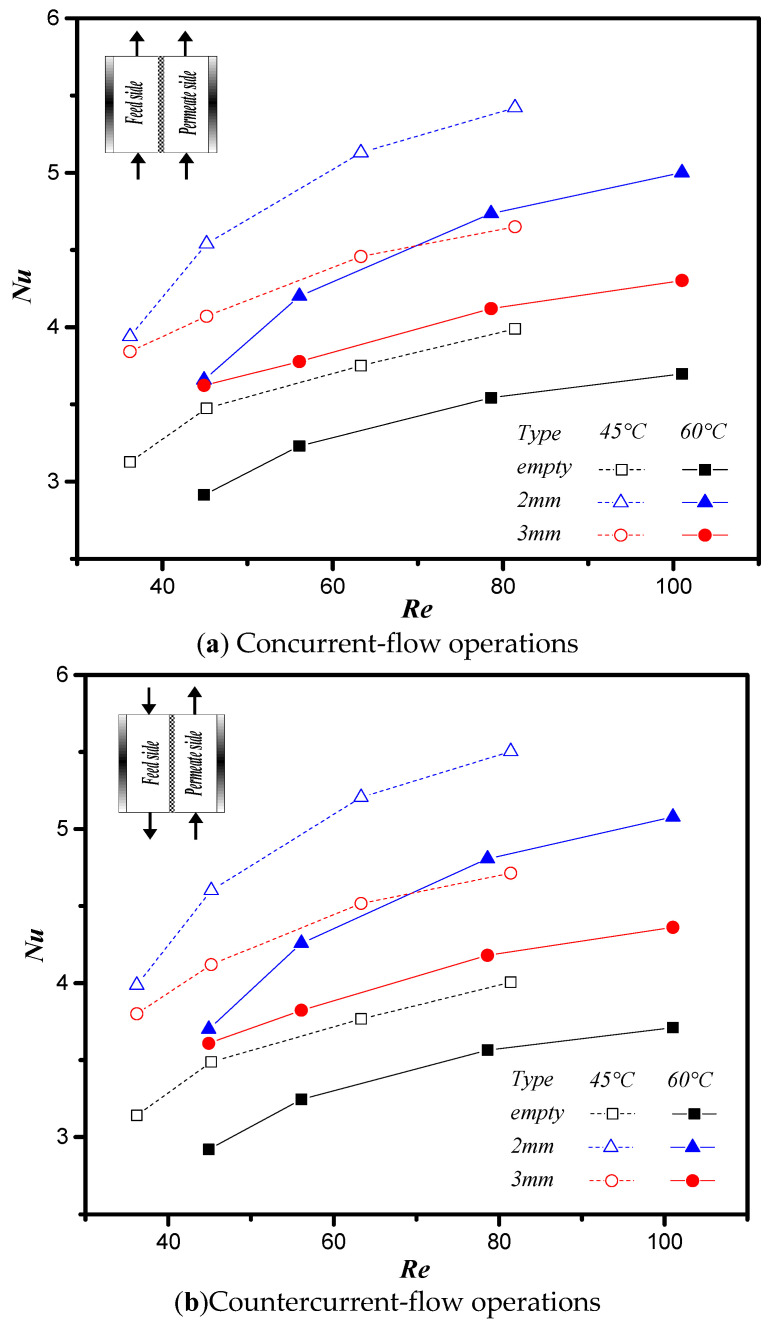
Comparisons of theoretical Nusselt numbers of two operations. Dependence of *Nu* on *Re* (solid symbols: 60 °C, open symbols: 45 °C).

Figure 16 presents the dependence of the Nusselt number on the Reynolds number. The figure shows that the Nusselt number increases as the Reynold number and the inlet saline temperature increase under both concurrent- and countercurrent-flow operations. Notably, the insertion of the diagonal carbon-fiber spacers significantly increases the Nusselt number and the convective heat transfer rate for both 2 and 3 mm open-slot widths. Despite the effect on the flow pattern, the change in the spacer width from 2 to 3 mm only leads to a moderate effect on the change of the Nusselt number.

**Figure 17:**
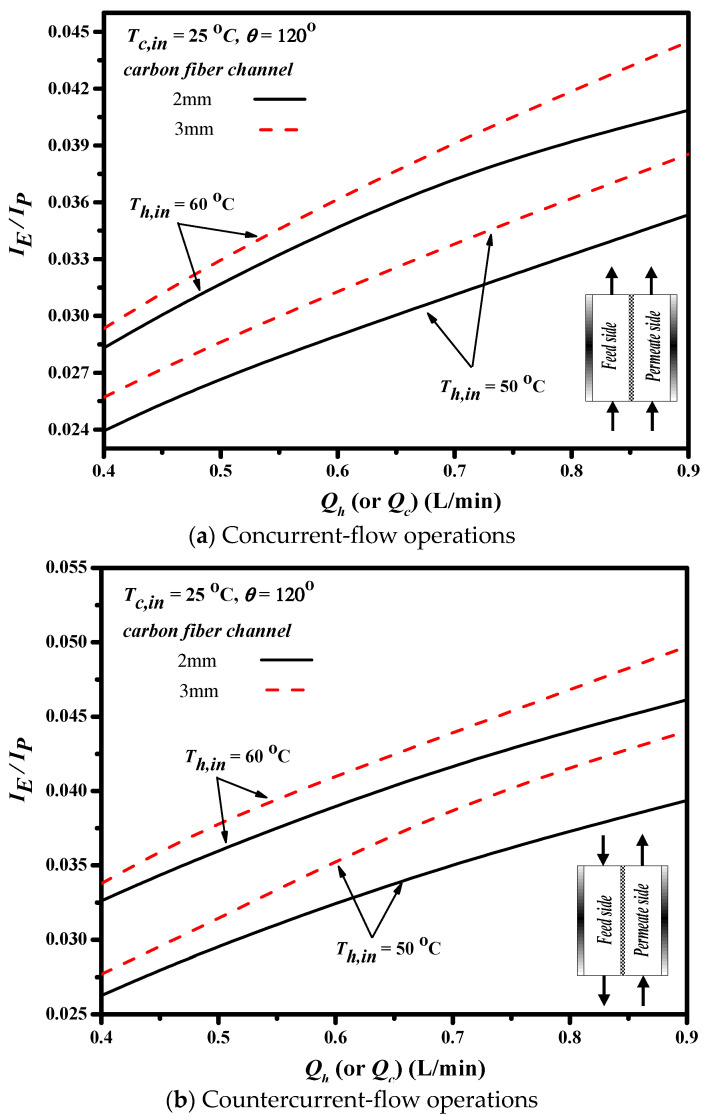
Effects of flow patterns on the value of $I_{E}/I_{P}$.

This study further examines the device performance by evaluating the desirable permeate flux increment to undesirable power consumption increment $I_{E}/I_{P}$ ratio due to inserting cross-diagonal carbon-fiber spacers in the flow channel associated with more flow resistance. The effects of flow configuration, carbon-fiber spacer widths and volumetric flow rate on $I_{E}/I_{P}$ are shown in Figure 17. The value of $I_{E}/I_{P}$ increases with increasing volumetric flow rate, which reveals that the expenses of energy consumption increment can compensate by the permeate flux enhancement. The value of $I_{E}/I_{P}$ in countercurrent-flow configuration with more significant temperature gradient is higher than that in concurrent-flow configuration due to utilizing the driving-force temperature gradient more effectively. The ratio of $I_{E}/I_{P}$ of the channel with 3 mm carbon-fiber spacers is higher than that of the channel with 2 mm carbon-fiber spacers. In other words, inserting 3 mm carbon-fiber spacer gives a higher value of $I_{E}/I_{P}$, which reflects that a more effective operation in increasing the permeate flux at the expense of energy consumption is expected. Although the permeate flux and the Nusselt number of the channel with 2 mm carbon-fiber spacers in Figure 13, Figure 14 and Figure 16 are higher than that of the channel with 3 mm carbon-fiber spacers, the energy consumption of the former channel is also higher. Comparisons of the economic feasibility among DCMD modules with inserting different widths of cross-diagonal carbon-fiber spacers were examined under both design and operating conditions.

## 6. Conclusions

A parallel-plate direct contact membrane distillation module with net-like cross-diagonal carbon-fiber spacers to enhance the permeate flux was investigated theoretically and experimentally. The theoretical predictions of the permeate flux enhancement by inserting turbulence promoters of cross-diagonal carbon-fiber spacers for various hot feed flow rates, inlet saline temperature and the carbon-fiber spacer widths under both concurrent- and countercurrent-flow operations were examined, and the correlated expression of Nusselt number was obtained as well. Comparisons of the permeate flux enhancements were made and the following conclusions were drawn:

1. The permeate flux increases with the increase of the volumetric flow rate.
2. Higher inlet saline temperature yields higher permeate flux productivity.
3. The permeate flux enhancement is obtained by inserting net-like cross-diagonal carbon-fiber spacers where the enhancement of the 2 mm slot opening is higher than that of the 3 mm one.
4. The permeate flux increases with increasing hydrodynamic angle in the slot of the cross-diagonal carbon-fiber spacers.
5. A maximum of 45.1% permeate flux enhancement was found in the device with cross-diagonal carbon-fiber spacers compared to that in the empty channel device under countercurrent-flow operations of 120° hydrodynamic angle.
6. A more considerable permeate flux was achieved in countercurrent-flow operations than in concurrent-flow operations due to the larger temperature gradient for countercurrent-flow operations.
7. The economic consideration of $I_{E}/I_{P}$ for permeate flux enhancement to power consumption increment concluded that the power utilization is more effective for the channel with cross-diagonal carbon-fiber spacers in higher hot saline water flow rate, and the ratio of $I_{E}/I_{P}$ for the 3 mm slot opening is higher than that of the 2 mm one.

A new contribution of this study is the desirable effect of raising the turbulence intensity as an alternative strategy [10] on the permeate flux in a direct contact membrane distillation module. The advantage of this membrane distillation device is it is somewhat easier to implement the experimental setup and has lower production costs. The alternative configurations of carbon-fiber spacers require further investigations to derive the optimal ratio between the permeate flux enhancement and energy consumption increment to make good economic sense.

## Figures and Tables

**Table 1:** (**a**) Comparison of theoretical and experimental permeate fluxes for concurrent flow. (**b**) Comparison of theoretical and experimental permeate fluxes for countercurrent flow.

$T_{h,\;in}$ (°C)	$Q_{h}\times 10^{6}$ (m^3^ s^−1^)	**(a) Cross-Diagonal Carbon-Fiber Spacers (2 mm)**								
		60°			90°				120°	
		$N_{\exp}^{\prime \prime }\times 10^{3}$ kg m^−2^ s^−1^	$N_{theo}^{\prime \prime }\times 10^{3}$ kg m^−2^ s^−1^	E(%)	$N_{\exp}^{\prime \prime }\times 10^{3}$ kg m^−2^ s^−1^	$N_{theo}^{\prime \prime }\times 10^{3}$ kg m^−2^ s^−1^	E(%)	$N_{\exp}^{\prime \prime }\times 10^{3}$ kg m^−2^ s^−1^	$N_{theo}^{\prime \prime }\times 10^{3}$ kg m^−2^ s^−1^	E(%)
45	6.67	0.69	0.74	6.33	0.69	0.75	8.18	0.72	0.77	5.77
	8.33	0.80	0.83	3.90	0.80	0.85	5.09	0.80	0.80	7.54
	11.7	0.89	0.97	8.12	0.93	0.98	5.60	0.99	1.00	1.01
	15.0	1.00	1.09	8.08	1.06	1.10	3.82	1.11	1.12	1.62
50	6.67	0.88	0.96	8.37	0.89	0.98	8.56	0.91	0.99	8.78
	8.33	1.05	1.10	4.64	1.10	1.12	1.37	1.15	1.14	0.94
	11.7	1.29	1.30	0.69	1.36	1.32	3.49	1.42	1.35	5.23
	15.0	1.44	1.46	1.25	1.46	1.48	0.94	1.56	1.50	4.51
55	6.67	1.09	1.20	8.90	1.10	1.22	9.87	1.15	1.26	9.26
	8.33	1.29	1.39	7.67	1.33	1.45	8.01	1.40	1.47	5.24
	11.7	1.54	1.68	8.18	1.59	1.71	7.27	1.68	1.75	4.06
	15.0	1.80	1.90	5.53	1.82	1.93	5.74	2.06	1.98	4.00
60	6.67	1.41	1.55	9.04	1.44	1.59	9.56	1.50	1.62	7.65
	8.33	1.64	1.79	8.56	1.67	1.83	8.54	1.75	1.86	6.21
	11.7	2.13	2.09	1.77	2.23	2.13	4.77	2.25	2.19	2.92
	15.0	2.37	2.38	0.24	2.49	2.43	2.46	2.52	2.46	2.46
$T_{h,\;in}$ (°C)	$Q_{h}\times 10^{6}$ (m^3^ s^−1^)	**(b) Cross-Diagonal Carbon-Fiber Spacers (2 mm)**								
		60°			90°				120°	
		$N_{\exp}^{\prime \prime }\times 10^{3}$ kg m^−2^ s^−1^	$N_{theo}^{\prime \prime }\times 10^{3}$ kg m^−2^ s^−1^	E(%)	$N_{\exp}^{\prime \prime }\times 10^{3}$ kg m^−2^ s^−1^	$N_{theo}^{\prime \prime }\times 10^{3}$ kg m^−2^ s^−1^	E(%)	$N_{\exp}^{\prime \prime }\times 10^{3}$ kg m^−2^ s^−1^	$N_{theo}^{\prime \prime }\times 10^{3}$ kg m^−2^ s^−1^	E(%)
45	6.67	0.74	0.81	8.74	0.76	0.83	8.75	0.79	0.85	6.79
	8.33	0.84	0.92	9.04	0.87	0.94	7.38	0.91	0.96	5.55
	11.7	1.04	1.06	2.55	1.07	1.07	0.33	1.15	1.10	4.49
	15.0	1.14	1.18	3.26	1.23	1.19	2.66	1.26	1.22	3.66
50	6.67	0.97	1.06	8.50	9.78	1.07	8.63	1.04	1.10	5.56
	8.33	1.14	1.26	9.26	1.27	1.27	0.12	1.32	1.31	1.31
	11.7	1.44	1.47	1.69	1.52	1.46	4.09	1.58	1.52	3.82
	15.0	1.68	1.62	3.49	1.74	1.64	5.87	1.80	1.66	8.00
55	6.67	1.29	1.41	8.86	1.34	1.45	7.77	1.36	1.50	9.37
	8.33	1.48	1.62	8.73	1.55	1.69	7.86	1.68	1.72	2.60
	11.7	1.80	1.90	5.60	1.85	1.94	4.82	1.95	1.99	1.73
	15.0	2.12	2.07	2.42	2.14	2.10	2.08	2.25	2.16	3.81
60	6.67	1.70	1.79	5.27	1.71	1.83	6.89	1.78	1.88	4.88
	8.33	1.94	2.07	6.68	1.98	2.11	6.44	2.07	2.15	4.03
	11.7	2.51	2.35	6.73	2.54	2.40	5.69	2.51	2.46	2.29
	15.0	2.71	2.66	1.85	2.75	2.70	1.97	2.79	2.73	2.07

**Table 2:** (**a**) Effects of hydrodynamic angles on flux enhancement for concurrent flow. (**b**) Effects of hydrodynamic angles on flux enhancement for countercurrent flow.

**(a) Effects of Hydrodynamic Angles on Flux Enhancement for Concurrent Flow**										
$T_{h,\;in}$ (°C)	$Q_{h}\times 10^{6}$ (m^3^ s^−1^)	Empty Channel	2 mm				3 mm			
			60°		90°		120°		120°	
		$N_{theo}^{\prime \prime }\times 10^{3}$ kg m^−2^s^−1^	$N_{theo}^{\prime \prime }\times 10^{3}$ kg m^−2^s^−1^	$I_{E}$	$N_{theo}^{\prime \prime }\times 10^{3}$ kg m^−2^s^−1^	$I_{E}$	$N_{theo}^{\prime \prime }\times 10^{3}$ kg m^−2^s^−1^	$I_{E}$	$N_{theo}^{\prime \prime }\times 10^{3}$ kg m^−2^s^−1^	$I_{E}$
50	6.67	0.81	0.96	18.7	0.98	20.4	0.99	22.6	0.97	19.8
	8.33	0.91	1.10	21.6	1.12	23.7	1.14	25.6	1.10	21.7
	11.7	1.03	1.30	26.3	1.32	28.0	1.35	30.7	1.28	24.6
	15.0	1.11	1.46	31.4	1.48	33.1	1.50	34.7	1.43	29.0
60	6.67	1.27	1.55	21.7	1.59	25.1	1.62	27.8	1.54	21.3
	8.33	1.43	1.79	25.1	1.83	27.8	1.86	30.3	1.78	24.2
	11.7	1.60	2.09	30.1	2.13	33.2	2.19	36.9	2.07	29.6
	15.0	1.75	2.38	35.6	2.43	38.6	2.46	40.0	2.35	34.1
**(b) Effects of Hydrodynamic Angles on Flux Enhancement for Countercurrent Flow**										
$T_{h,\;in}$ (°C)	$Q_{h}\times 10^{6}$ (m^3^ s^−1^)	Empty channel	2 mm				3 mm			
			60°		90°		120°		120°	
		$N_{theo}^{\prime \prime }\times 10^{3}$ kg m^−2^ s^−1^	$N_{theo}^{\prime \prime }\times 10^{3}$ kg m^−2^ s^−1^	$I_{E}\;(\%)$	$N_{theo}^{\prime \prime }\times 10^{3}$ kg m^−2^ s^−1^	$I_{E}$(%)	$N_{theo}^{\prime \prime }\times 10^{3}$ kg m^−2^ s^−1^	$I_{E}$ (%)	$N_{theo}^{\prime \prime }\times 10^{3}$ kg m^−2^ s^−1^	$I_{E}$(%)
50	6.67	0.88	1.06	21.4	1.07	22.2	1.10	25.9	1.06	21.0
	8.33	1.01	1.26	24.6	1.27	26.1	1.31	29.2	1.25	23.7
	11.7	1.13	1.47	29.7	1.46	29.4	1.52	34.7	1.46	29.5
	15.0	1.20	1.62	35.0	1.64	36.6	1.66	38.7	1.60	33.2
60	6.67	1.42	1.78	25.0	1.83	29.0	1.88	32.1	1.78	25.5
	8.33	1.59	2.05	28.9	2.10	31.9	2.15	35.5	2.05	28.7
	11.7	1.74	2.35	35.3	2.40	38.9	2.46	41.1	2.32	33.1
	15.0	1.88	2.66	41.7	2.70	43.6	2.73	45.1	2.58	37.3

## Abbreviations

$a_{w}$	Water activity in NaCl solution
$C_{p,c}$	Heat capacity of cold fluid (J kg^−1^ K^−1^)
$C_{p,h}$	Heat capacity of hot fluid (J kg^−1^ K^−1^)
$c_{m}$	Mass transfer coefficient of membrane (kg m^−2^ Pa^−1^ s^−1^)
D	Equivalent hydraulic diameter of empty channel (m)
$D_{h,h}$	Equivalent hydraulic diameter of hot side (m)
$D_{h,c}$	Equivalent hydraulic diameter of cold side (m)
d	Height of flow channel (m)
*d_p_*	Height of carbon-fiber spacers
*E*	Deviation of experimental results from the theoretical predictions
$f_{F}$	Fanning friction factor
$h_{c}$	Convection coefficient of cold fluid (W m^−2^ K^−1^)
$h_{h}$	Convection coefficient of hot fluid (W m^−2^ K^−1^)
$H_{i}$	Hydraulic dissipate energy (J kg^−1^), i=promoter,empty
$H_{m}$	Thermal convection coefficient of membrane (W m^−2^ K^−1^)
$I_{E}$	Increased percentage of permeate flux
$I_{P}$	Raised percentage of hydraulic loss
L	Axial distance (m)
k	Thermal conductivity coefficient of hot saline feed (W m^−1^ K^−1^)
$k_{g}$	Thermal conductivity coefficient of the vapor in the membrane pore (W m^−1^ K^−1^)
$k_{s}$	Thermal conductivity coefficient of the solid membrane material (W m^−1^ K^−1^)
ℓwf	Friction loss of conduits (J kg^−1^)
$M_{W}$	Molecular weight of water (kg mol^−1^)
$\dot{m}$	Mass flow rate (kg s^−1^)
$N^{\prime \prime }$	Permeate flux (kg m^−2^ h^−1^)
$Nu_{}$	Nusselt number
$Nu^{E}$	Nusselt number of the turbulence promoter
$Nu_{lam}$	Dimensionless Nusselt number for laminar flow
P	Pressure (Pa)
$P_{1}^{sat}$	Saturation vapor pressure in the cold feed flow side (Pa)
$P_{2}^{sat}$	Saturation vapor pressure in the hot feed flow side (Pa)
$P_{w}{}^{sat}$	Saturated vapor pressure of pure water (Pa)
Pr	Prandtl number
${q^{\prime \prime }}_{}$	Heat transfer rate (W/m^2^)
${q^{\prime \prime }}_{c}$	Heat transfer rate between cooling plate and cold fluid (W/m^2^)
${q^{\prime \prime }}_{h}$	Heat transfer rate between hot fluid and membrane surface (W/m^2^)
${q^{\prime \prime }}_{m}$	Heat transfer rate between membrane surface of hot fluid and air gap (W/m^2^)
Q	Volumetric flow rate (m^3^ s^−1^)
R	Gas constant (8.314 J mol^−1^ K^−1^)
*Re*	Reynolds number
T	Temperature (°C)
$T_{m}$	Mean temperature in membrane (°C)
$\bar{v}$	Average velocity (m s^−1^)
W	Width of flow channel
We	Width of carbon-fiber spacers
${\left|Y_{m}\right|}_{\ell n}$	Natural log mean mole fraction of air
$x_{NaCl}$	Liquid mole fraction of NaCl
$x_{w}$	Liquid mole fraction of water
z	Axial coordinate along the flow direction (m)
Greek letters	
$\alpha^{E}$	Heat transfer enhancement factor
β	Aspect ratio of the channel
ΔP	Vapor pressure difference of membrane (Pa)
$\delta_{m}$	Thickness of membrane (µm)
ε	Membrane porosity
$\varepsilon_{e}$	Channel voidage
λ	Latent heat of water (J/kg)
μ	Fluid viscosity (kg s^−1^ m^−1^)
ρ	Density (kg m^−3^)
$T_{PC\;}$	Temperature polarization coefficients
Subscripts	
1	Membrane surface on cold feed side
2	Membrane surface on hot feed side
*h*	In the hot feed flow channel
*c*	In the cold feed flow channel
*carbon fiber*	Inserting cross-diagonal carbon-fiber spacers
*empty*	Inserting nylon fiber as supporters
*exp*	Experimental results
*in*	Inlet
*lam*	Empty channel
*out*	Outlet
*theo*	Theoretical predictions
Superscripts	
*E*	The channel with inserting cross-diagonal carbon-fiber spacers
